# ﻿A peculiar new species of Dione (Agraulis) Boisduval & Le Conte (Lepidoptera, Nymphalidae, Heliconiinae) associated with *Malesherbia* Ruiz & Pavón (Passifloraceae) in xeric western slopes of the Andes

**DOI:** 10.3897/zookeys.1113.85769

**Published:** 2022-07-18

**Authors:** Jackie Farfán, José Cerdeña, Héctor A. Vargas, Gislene L. Gonçalves, Gerardo Lamas, Gilson R. P. Moreira

**Affiliations:** 1 PPG Biologia Animal, Departamento de Zoologia, Instituto de Biociências, Universidade Federal do Rio Grande do Sul, Av. Bento Gonçalves, 9500, Porto Alegre, RS 91501-970, Brazil; 2 Museo de Historia Natural, Universidad Nacional de San Agustín de Arequipa, Av. Alcides Carrión s/n, Arequipa, Peru; 3 Departamento de Recursos Ambientales, Facultad de Ciencias Agronómicas, Universidad de Tarapacá, Casilla 6-D, Arica, Chile; 4 Departamento de Genética, Instituto de Biociências, Universidade Federal do Rio Grande do Sul, Av. Bento Gonçalves 9500, Porto Alegre RS, 91501-970, Brazil; 5 Departamento de Entomología, Museo de Historia Natural, Universidad Nacional Mayor de San Marcos, Apartado 14-0434, Lima-14, Peru; 6 Departamento de Zoologia, Instituto de Biociências, Universidade Federal do Rio Grande do Sul, Av. Bento Gonçalves 9500, Porto Alegre RS, 91501-970, Brazil

**Keywords:** Chile, Heliconiines, immature stages, Peru, taxonomy

## Abstract

Butterflies associated with xerophytic environments of the Andes have been little studied, and they exhibit high levels of endemism. Herein Dione (Agraulis) dodona Lamas & Farfán, **sp. nov.** (Nymphalidae; Heliconiinae) is described, distributed on the western slopes of the Andes of Peru and northern Chile, between 800 and 3,000 m elevation. Adults of both sexes, and the immature stages, are described and illustrated based on light and scanning electron microscopy. The immature stages are associated with *Malesherbiatenuifolia*D. Don (Passifloraceae) found in xeric environments, representing a new record of this genus as a host plant for the subfamily Heliconiinae. Conspicuous morphological differences are presented for all stages at the generic level. Based on a phylogenetic analysis of the COI barcode mitochondrial gene fragment, D. (A.) dodona Lamas & Farfán, **sp. nov.** is distinguished as an independent lineage within the *Agraulis* clade of *Dione*, with ca. 5% difference to congeneric species.

## ﻿Introduction

The Andes are a ~ 8000 km long mountain belt on western South America, forming one of the longest mountain ranges on Earth (Montgomery et al. 2001), and harboring a variety of ecosystems with different environmental characteristics (Myers et al. 2000; Veblen et al. 2007). The western slopes of the central Andes join the Peru-Chile Pacific and Atacama deserts from 10 °S in central Peru to 30 °S in northern Chile, where xerophytic shrub land vegetation is predominant between 1,500 and 3,000 m elevation (Gutiérrez et al. 1998; Montesinos et al. 2012), containing many endemic plants and animals (Zeballos-Patrón et al. 2001; Arakaki and Cano 2003; Gutiérrez et al. 2019; Farfán et al. 2020; Málaga et al. 2020). The butterfly fauna in this area is depauperate and poorly known, with very few species recorded among the Papilionidae, Hesperiidae, Pieridae, and Nymphalidae, including four species of passion-vine butterflies (Heliconiinae) previously ascribed to *Dione* Hübner, [1819] and *Agraulis* Boisduval & Le Conte, [1835] (Peña and Ugarte 2006; Benyamini et al. 2014; Farfán 2018).

Heliconiinae have been the subject of widespread study in various aspects of biology for the last 160 years, comprising more than 700 scientific publications (Bates 1862; Jiggins 2017), highlighting their importance as a model in the study of evolution (e.g., Mallet and Joron 1999), ecology (e.g., Gilbert 1991; Schluter 2000) and behavior (Deinert et al. 1994), among others. The phylogenetic relationships of the members of the Heliconiinae have been analyzed from a morphological (Penz 1999; Penz and Peggie 2003) and molecular (Brower and Egan 1997; Beltrán et al. 2002, 2007; Kozak et al. 2015) perspective, recognizing four tribes: Acraeini, Heliconiini, Argynnini and Vagrantini. The Heliconiini included until recently the genera *Philaethria* Billberg, 1820, *Podotricha* Michener, 1942, *Dryas* Hübner, [1807], *Dryadula* Michener, 1942, *Dione* Hübner, [1819], *Agraulis* Boisduval & Le Conte, [1835], *Eueides* Hübner, 1816, and *Heliconius* Kluk, 1780, of which the latter is the most diverse with ~ 45 species and 200 subspecies (Lamas 2004; Kozak et al. 2015; Zhang et al. 2019). All the heliconiine species have as host plants members of the Passifloraceae s. l. (Bremer et al. 2009), mainly *Passiflora* L. (de Castro et al. 2018). There is a large number of studies related to the coevolution between butterflies and host plants, using the *Passiflora*-heliconiine system as a model for the study of coevolution in insects (Ehrlich and Raven 1964; Gilbert 1972; Benson et al. 1975; de Castro et al. 2018).

Phylogenetic studies have proposed that *Agraulis* is among the oldest lineages within the Heliconiini (Brower and Egan 1997; Penz 1999; Beltrán et al. 2007). For a long time, it was considered as a monotypic genus, with eight subspecies, occupying the largest distribution area within the tribe, ranging from the central west of the USA to the center of Argentina and Chile (Núñez et al. 2022). Recently, Zhang et al. (2019) through genome-scale phylogenetic analyses suggested that Agraulis is best treated as a subgenus of Dione. However, later on, Núñez et al. (2022) based on morphometric and molecular data maintained the two separate genera as valid. They also raised the eight subspecies of *Agraulis* to the species level including an undescribed species from the western slope of the Andes, previously listed as *Agraulis* n. sp. in the checklist of Neotropical butterflies (Lamas 2004). The proposal of Zhang et al. (2019) is followed in this study.

This undescribed species has been considered rare, with only seven museum specimens known until 2013. The first one was collected in northern Chile in 1951 at an altitude of 3,000 m, and erroneously identified as *A.vanillae* (Linnaeus, 1758), as illustrated in the book Butterflies of Chile (Peña and Ugarte 2006). The additional specimens are known from central and southern Peru, and northern Chile. During a recent exploration in the Department of Arequipa, southern Peru, adults of this undescribed species were observed flying, crossing the road in a sector of a desert hillside with very sparse vegetation, and laying eggs on the flowers of *Malesherbiatenuifolia*D. Don (Passifloracae). Thus, by finding the host plant, and then rearing the immatures, it was possible to obtain sufficient material to carry out a comparative study at the generic level, where we confirmed for all stages that it does not belong to any described species of Dione (Agraulis).

Thus, in the present paper, the new species is described and illustrated based on the morphology of adults and immature stages. We also present a phylogenetic analysis of mitochondrial (COI) DNA sequences including congeneric species.

## ﻿Materials and methods

Immature stages were collected from plants of *Malesherbiatenuifolia*D. Don (Passifloraceae), near the village of Pacaychacra (15°50'57"S, 72°38'9"W), Condesuyos Province, 23–24 km SE from the town of Chuquibamba, Department of Arequipa, southern Peru, at 1,800 m elevation. They were brought to the laboratory of Área de Entomología, Museo de Historia Natural de la Universidad Nacional de San Agustin de Arequipa (**MUSA**), and maintained under natural conditions of temperature and humidity. The eggs were placed until larval hatching. Fresh larvae were transferred to small plastic containers and fed with *M.tenuifolia* leaves. Six larvae successfully completed development to adults, with four males and two females emerging that were deposited in the collection of the MUSA. Additional field-collected eggs, larvae and pupae were fixed in Dietrich´s fluid and preserved in 70% ethanol. Also, flying adults were eventually netted near the host plants. Studies of morphology of the immature stages were conducted at the Laboratório de Morfologia e Comportamento de Insetos (**LMCI**), Departamento de Zoologia, Universidade Federal do Rio Grande do Sul (**UFRGS**), Porto Alegre, RS, Brazil.

Adult specimens of related taxa were examined at Museo de Historia Natural, Universidad Nacional Mayor de San Marcos, Lima, Peru (**MUSM**), and at MUSA. Photographs of all relevant type specimens were examined at MUSM; such images are also available in Warren et al. (2017). Images taken of male genitalia dissected from the new species are presented in Suppl. material 1.

Genitalia dissections were performed using standard techniques, where abdomens of adults were previously soaked in hot 10% KOH solution for 10 min, and dissected parts were stored in glycerol. To study the venation, wings were diaphanized by soaking them in 2% NaClO aqueous solution (bleach), and then dry-mounted.

Morphological observations were performed with the aid of a Zeiss Stemi 305 stereo microscope, and structures selected to be illustrated were previously photographed with a Nikon DS-Ri2 camera through a Nikon SMZ25 stereo microscope at the Laboratório de Sistemática Animal of Universidad Nacional San Agustin de Arequipa (**UNSA**). Images were assembled and edited in Nikon NIS-Elements and Photoshop version 21.2.0. The descriptive terminology of morphological structures follows da Silva et al. (2006), Beebe et al. (1960), Emsley (1963) and Klots (1970), for eggs, larvae, pupae, and adults, respectively.

For scanning electron microscope analyses, specimens were dehydrated in a BalTec CPD 030 critical-point dryer, mounted with double-sided tape on metal stubs, and coated with gold in a Quorum Q150R plus sputter coater. They were then examined and photographed in a XL-30 Philips scanning electron microscope at the Laboratório Central de Microscopia e Microanálise (LabCEMM) of Pontifícia Universidade Católica do Rio Grande do Sul (**PUCRS**), Porto Alegre, RS, Brazil. Priority in this case was given to key diagnostic characters that were used to distinguish the new *Dione* species from congeners; additional scanning electron micrographs are presented for the immature stages in Suppl. material 2.

Genomic DNA was extracted from two specimens of the new *Dione* species (Table 1), collected in the type locality, following the procedures described in Huanca-Mamani et al. (2015). A fragment of 650 base pairs of the COI gene was amplified by polymerase chain reaction (PCR) with the primers LEP-F1 and LEP-R1 (Hebert et al. 2004). PCR amplicons were purified and sequenced by Macrogen (Republic of South Korea) using LEP-F1 primer. Sequences obtained in this study were deposited in GenBank and BOLD databases (Table 1). The phylogenetic status of the new *Dione* species was explored by combining our sequences with COI data of six congeners (D. (A.) incarnata Riley, 1926, D. (A.) forbesi Michener, 1942, D. (A.) insularis (Maynard, 1889), D. (A.) lucina (C. Felder & R. Felder, 1862), D. (A.) maculosa Stichel, [1908], and D. (A.) vanillae (Linnaeus, 1758)) obtained from Núñez et al. (2022). In addition, three species of Dione (Dione), *juno* (Cramer, 1779), *glycera* (C. Felder & R. Felder, 1861) and *moneta* Hübner, [1825] were included in the analysis (Table 1). The COI-tree was inferred by using the Maximum Likelihood (ML) method and General Time Reversible model (Nei and Kumar 2000), with heuristic search obtained automatically by applying Neighbor-Join and BioNJ algorithms to a matrix of pairwise distances, with 500 bootstrap replications. Analysis was conducted in MEGA X (Kumar et al. 2018). Genetic distances between species of subgenera *Agraulis* and *Dione* were quantified using the Kimura 2-parameter model in MEGA.

**Table 1.** Specimens used for molecular analyses of Dione (Agraulis) dodona sp. nov. The collection sites (country/locality) and vouchers from which the sequences derived are presented, including the references. Genbank and BOLD identifiers link the record to the databases.

Species	Country/Locality	Voucher	Genbank/BOLD Accession code	Reference
Dione (Agraulis) dodona sp. nov.	Peru / Arequipa	J151	OM925454/BIGLE001-22	This study
Dione (Agraulis) dodona sp. nov.	Peru / Arequipa	459	OM925453 /BIGLE002-22	This study
Dione (Agraulis) incarnata	Costa Rica / Guanacaste	00-SRNP-16229	GU333737 / MHACG518-04	Núñez et al. 2022
Dione (Agraulis) forbesi	Peru	G3	MZ229712.1	Núñez et al. 2022
Dione (Agraulis) insularis	Dominican Republic	NW152-16	GQ864730	Núñez et al. 2022
Dione (Agraulis) lucina	Ecuador	LEP-58352	MZ229704.1	Núñez et al. 2022
Dione (Agraulis) maculosa	Argentina/ Entre Ríos	MACN-Bar-Lep-ct 01616	MF545390 / LEPAR178-11	Lavinia et al. 2017
Dione (Agraulis) vanillae	Ecuador	LEP-55200	MZ229702.1	Núñez et al. 2022
Dione (Dione) glycera	-	BMC17102	MN306819.1	Marín et al. 2021
Dione (Dione) juno	Peru/ San Martín	8727	KP074744.1	Kozak et al. 2015
Dione (Dione) moneta	Argentina/ Salta	MACN-Bar-Lep-ct 07589	MZ335918.1/LNOA484-16	NCBI deposit

Abbreviations for the museum collections and institutions from which specimens were examined are:

**LMCI**Laboratório de Morfologia e Comportamento de Insetos, Universidade Federal do Rio Grande do Sul, Porto Alegre, Rio Grande do Sul, Brazil;

**MHNS**Museo Nacional de Historia Natural de Chile, Santiago de Chile, Chile;

IDEA Colección Entomológica de la Universidad de Tarapacá, Arica, Chile;

**MUSA**Museo de Historia Natural, Universidad Nacional de San Agustín de Arequipa, Arequipa, Peru;

MUSM Museo de Historia Natural, Universidad Nacional Mayor San Marcos, Lima, Peru.

## ﻿Taxonomic account

### ﻿ Dione (Agraulis) dodona

Taxon classificationAnimaliaLepidopteraNymphalidae

Lamas & Farfán sp. nov.

D01F28A4-9632-52F6-AB26-5F05EEB24A53

https://zoobank.org/687E38BC-19C6-48F0-9B93-EE7B9A7C40A5

Figs 1 , 2 , 3 , 4 , 5 , 6 , 7 , 8 , 9 , 10

Dione vanillae : Peña 1951: 262.
Agraulis vanillae : Ureta 1963: 114–115; Pérez-D’Angello 1970: 6; Etcheverry 1970: 95; Peña and Ugarte 2006: 313, figs; Benyamini et al. 2014: 18.
Agraulis vanillae forbesi : Herrera,1972: 73.
Agraulis [n. sp.]: Lamas 2004: 264.
Agraulis sp. n.: Farfán 2018: 367.
Agraulis sp.: Núñez et al. 2022: 152–178.

#### Type locality.

Peru, Arequipa, Pacaychacra [15°54'S, 72°33'W], 1500 m.

#### Type material.

***Holotype*** ♂, Peru, Arequipa, Pacaychacra, 15°54'S, 72°33'W, 1500 m, reared from eggs collected on *Malesherbiatenuifolia* (Passifloraceae), 24.VII.2019, J. Farfán leg. deposited in MUSM. ***Paratypes*** (25♂, 17♀): Peru. **Lima**: 1♂, San Bartolomé, 1600 m, [11°55'S, 76°31'W], 21.iii.[19]81, P. Hocking (MUSM); 1♀, Cocachacra, 1450 m, [11°55'S, 76°32'W], 6.x.[19]83, P. Hocking [MUSM-ENT 008630] (MUSM); 1♀, Río Rímac, Chaute, 2350 m, 11°56'S, 76°30'W, 12.v.2012, P. Hocking (MUSM). **Arequipa**: 2♀, 7 km E Cháparra, 1450m, [15°41'S, 73°49'W], 14.iv.[19]88, G. Lamas [MUSM-ENT 008631, 008632] (MUSM); 1♂, entre Majes y Chuquibamba, 15°55'S, 72°33'W, 1500 m, 24.iv.2017, G. Lamas (MUSM); 1♂ same data as holotype [LMCI 357–51] (LMCI); 1♂ same data as holotype (MUSA); 1♂, 2♀, same data as holotype but with date 15.XII.2020 (MUSA); 1♂ same data as holotype but with date 24.vii.2021 (MUSA); 4♂ Aplao, Valle Majes, 15°53'46"S, 72°28'03"W, 800 m, 02.vi.2013, Leg. J. Cerdeña / M. Delgado (MUSA); 1♂, Condesuyos, Pacaychacra,15°54'59.2"S, 72°33'01.5"W, 1500 m, 02.IX.2020, Leg. Jose Cerdeña (MUSA); 1♂, same data, but 13.XII.2020 (MUSA); 1♀, same data, but 24.VII.2021, Jackie Farfán (MUSA); 1♀, same data, but 12.XI.2019, [LMCI 357–52] (LMCI); 3♂, Yura, 2 Km SW Yura viejo, 16°13'19"S, 71°42'23"W, 2600 m 21.IV.2022, Leg. Jackie Farfán (MUSA). **Moquegua**: 6♂, 1♀, Torata, 170630/705036 [17°06'30"S, 70°50'36"W], 2090m, 26.VII.2021, Leg. Jackie Farfán (MUSA); 1♂, 1♀, same data [LMCI 357–53, LMCI 357–54] (LMCI); 4♀, La Capilla, 13 Km S Puquina, 164450/711054 [16°44'50"S, 71°10'54"W], 1800 m, 28.XII.2013, Leg. J. Farfán / J. Cerdeña (MUSA); 1♀, Omate, 3 Km SW Omate, 16°41'15.27"S, 70°59'13.73"W, 2000 m, 27.X.2017, Leg. Robert Cornejo (MUSA). **Tacna**: 1♂, Chululuni, 17°22'02"S, 70°28'24"W, 1800 m, 18.XII.2020, Leg. Jackie Farfán (MUSA). CHILE. **Arica**: 1♂, Las Peñas, 18°33'08"S, 69°46'03"W, 1580 m, 02.XII.2020, H. A. Vargas leg. [IDEA-LEPI–2022–008] (IDEA); 1♀, same data, but 10.XI.2020; [IDEA-LEPI–2022–007] (IDEA). **Tarapacá**: 1♀, Iquique, Parca, 3000 m, [20°01'S, 69°01'W], ix/x.[19]51, L. E. Peña (MUSM); 1♂, Q[uebrada] de Guatacondo, “Cauquenisca” [= Cautenicsa], 2300 m, [20°56'S, 69°01'W], 26-X-1968, P. Millas (MHNS).

Immature stages preserved in 70% ethanol, with the same data as the holotype, collected on *Malesherbiatenuifolia* (Passifloraceae) with dates VI.2018, X.2019 and IV.2021, were deposited in LMCI, under accession numbers 357–31 (14 eggs), 357–33, 357–40, 357–44 (20 larvae), 357–46, 357–47, 357–48, 357–49, 357–50 (5 pupae).

#### Diagnosis.

Dione (Agraulis) dodona sp. nov. can be easily distinguished from its congeners by the wing pattern, presenting a black postdiscal spot between M_3_-A_1_ veins on the dorsal hindwing that is absent in all other species, and, also, by presenting a divided or partially divided silver spot in the discal cell on the ventral hindwing, always undivided in other species of Dione (Agraulis). In the male genitalia, the valvae have a rounded termen, without denticles, and the distal portion of the crista is narrow and straight. In other species, the termen is sub-triangular and shows denticles on the margin, and the distal apex of the crista is transversally enlarged. Also, the juxta has its upper edge slightly split in *dodona*, which is widely open in other species. The aedeagus is straight in lateral view without cornuti in *dodona*, up-curved near distal end with cornuti in other species. The female genitalia possess evenly wide signa in the proximal portion, composed of robust spines, unlike other species that have smaller spines; the proximal apex of signa is narrower and progressively enlarges distally.

Furthermore, the immature stages of Dione (Agraulis) dodona sp. nov. show differences with the available data compared to other *Agraulis* species (Beebe et al. 1960; Brown 1981; da Silva et al. 2006). In the egg, the number of horizontal carinae almost doubles the number described for Dione (Agraulis) maculosa [cited as ‘*A.vanillae*´] (11–13 vs. 17–19), the egg being taller in Dione (Agraulis) dodona sp. nov. In relation to the larval stage, in the first instar, the main difference is the size of the D2 setae in the abdominal segments, being very small in *maculosa* and *insularis* (1/4 the length of D1) (Beebe et al. 1960; da Silva et al. 2006), but reaching more than half the length of D1 in Dione (Agraulis) dodona sp. nov. (Fig. 9B). In the fifth instar, the head scoli in *maculosa* are well developed, whereas Dione (Agraulis) dodona sp. nov. bears short stout scoli (Figs 5D, 6E). Furthermore, the prothoracic dorsal plate in Dione (Agraulis) dodona sp. nov. has spine-like setae on top of enlarged conical projection (Fig. 9C), unlike *maculosa*, which has a simple seta bearing on a small projection (da Silva et al. 2006). In the pupa, the main differences are related to the head protuberances, which are small in Dione (Agraulis) dodona sp. nov. (Fig. 9D), more conspicuous, and as long as half the length of head in *maculosa*; also, the meso-dorsal crest is less pronounced in Dione (Agraulis) dodona sp. nov. (Fig. 9E), in *maculosa* the dorsal margins are more enlarged; protuberances on the abdominal segments do not occupy the entire length of the segment in Dione (Agraulis) dodona sp. nov. (Fig. 9F), in contrast to *maculosa* where protuberances fill all the length of the third abdominal segment.

#### Description.

**Figure 1.**
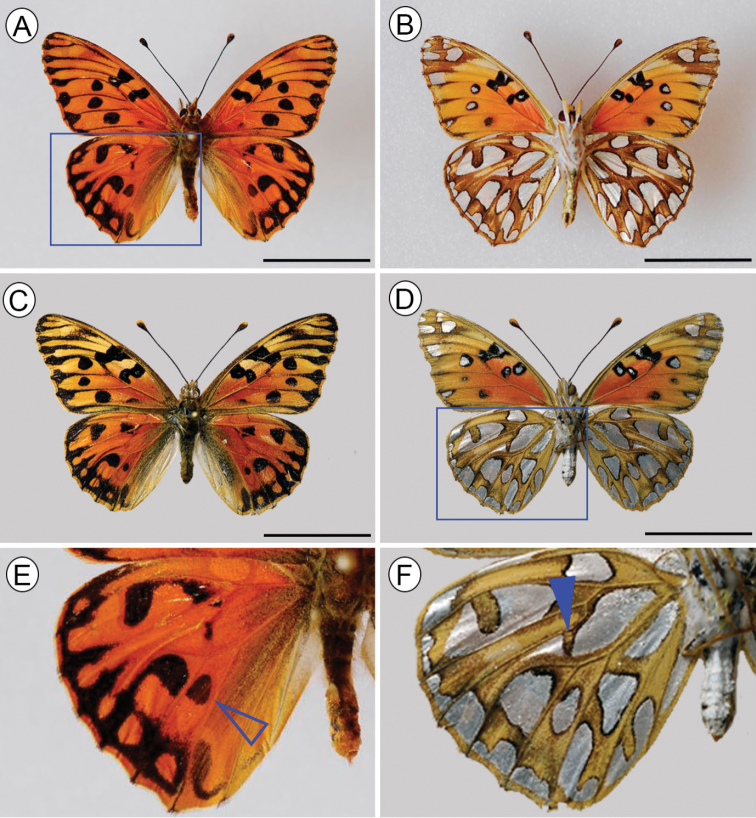
Adults of Dione (Agraulis) dodona sp. nov. **A, B** male, holotype **C, D** female paratype **E, F** details of hindwing pattern color (indicated by blue squares in **A** and **D** respectively), open arrow points black spot on Cu_1_-Cu_2_ cell, and close arrow on silver discal spot. Left: dorsal view; right: ventral view. Scale bars: 2 cm.

**Figure 2.**
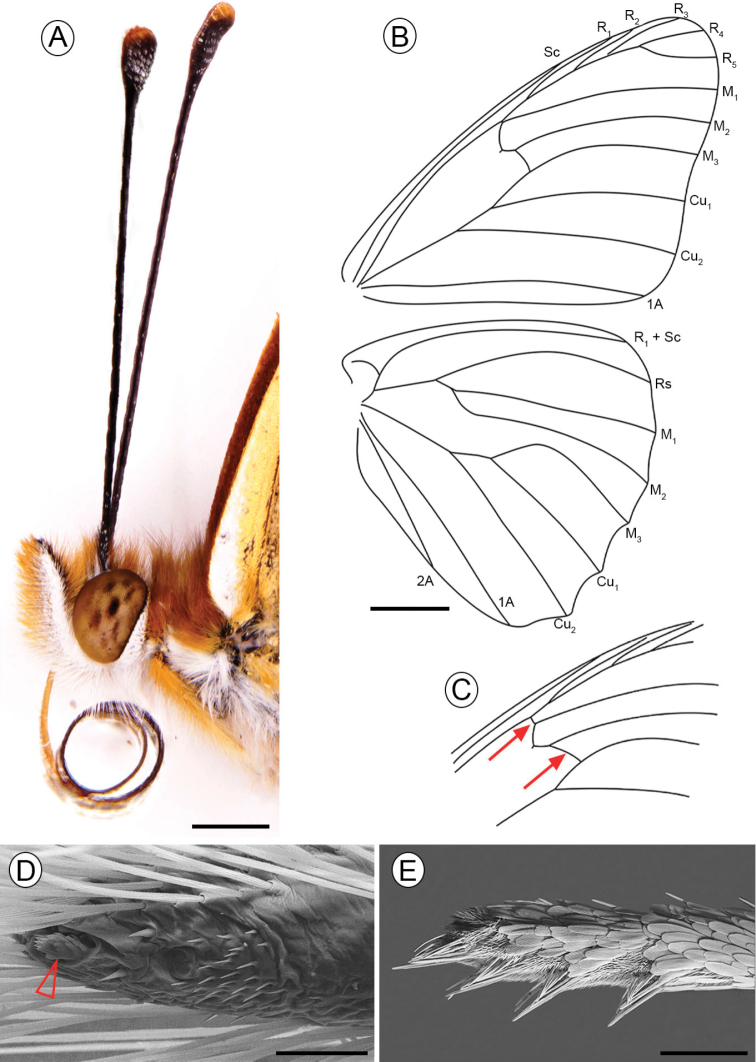
Adult morphology of Dione (Agraulis) dodona sp. nov. **A** head, male, lateral view **B** wing venation, male **C** detail of forewing venation, female (red setae points differences with male) **D, E** distal portion of prothoracic tarsi under scanning electron microscopy, male in ventral view **D** female in lateral view **E** distal tarsomere indicated by open arrow in E. Scale bars: 1 mm (**A**); 5 mm (**B**); 50 µm (**D**); 250 µm (**E**).

**Figure 3.**
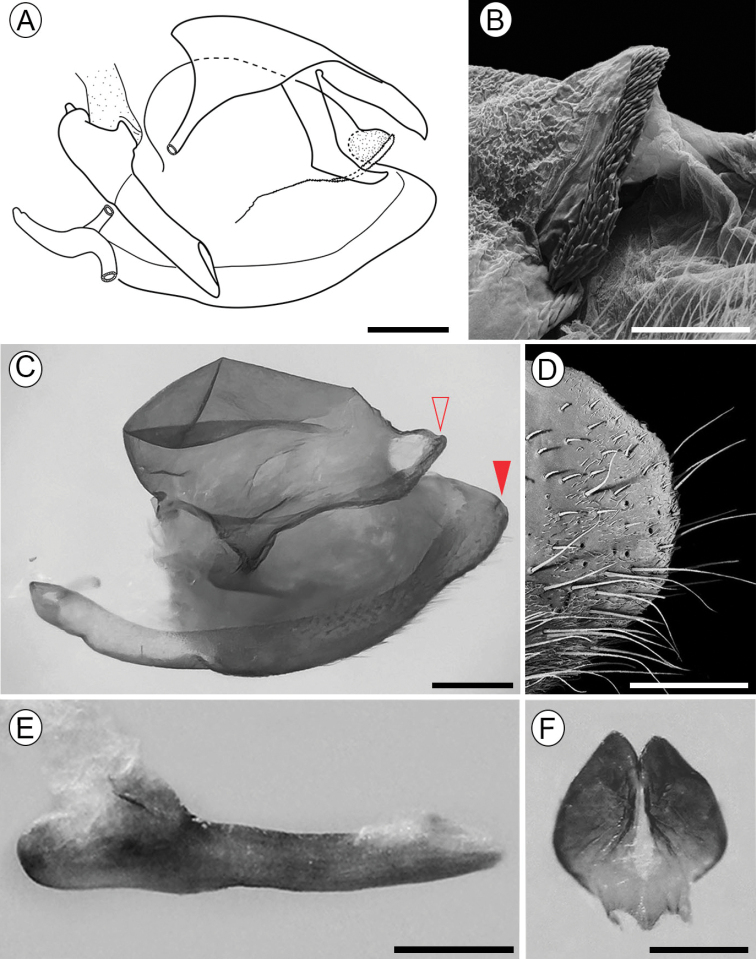
Male genitalia of Dione (Agraulis) dodona sp. nov. **A** general, mesal view **B** distal portion of valve crista under scanning electron microscopy (pointed with open arrow in **C**) **C** right valve **D** termen of valve under scanning electron microscopy (pointed with closed arrow in **C**) **E** aedeagus, lateral **F** juxta, posterior. Scale bars: 1mm (**A**); 250 µm (**B**); 500 µm (**C, E, F**); 200 µm (**D**).

**Figure 4.**
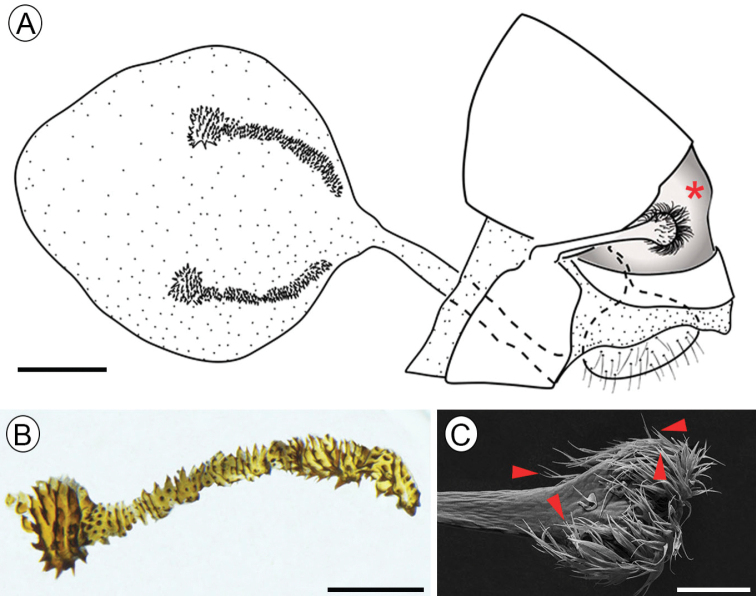
Female genitalia of Dione (Agraulis) dodona sp. nov. **A** external view, lateral **B** signum **C** stink club under scanning electron microscopy, specialized scales indicated by closed arrows. Asterisk indicated dorsal glands. Scale bars: 1 mm (**A**); 500 µm (**B**); 200 µm (**C**).

***Adult*.** (Figs 1–4). **Male**: Wingspan 44–52 mm (holotype 50 mm). *Head*: antennae approximately two thirds of the forewing in length, black with the tip of club orange (Fig. 2A), with 36 antennomers, 11 of which define the club. Palpus elongated, approximately twice the size of the head, with a dark brown dorsal color with light orange hairs, in ventral view white covered by white and orange hairs (Fig. 2A). *Thorax*: Generally brown. Body dorsally black with brown and orange hairs, ventrally covered by white and light orange scales, legs dorsally light orange with white and orange hairs at the base, ventrally white. Forewing length 25–28 mm (holotype: 27 mm), hindwing length 18–20 mm. Wing venation as described in Michener (1942) (Fig. 2B). Wing color pattern typical for the *Agraulis* clade except a black postdiscal spot between veins M_3_-A_1_ on dorsal hindwing and a silver spot located in the discal cell on ventral hindwing divided or partially divided (Fig. 1E, AF). *Abdomen*: dorsally brown with orange hairs, ventrally covered by white scales.

***Male genitalia***: Rounded and subtriangular valvae occupying most of the genital capsule, being wide anteriorly and narrowest in the apex, with rounded pointed apex with hairs on ventral margin (Fig. 3D), costa with eversible pouch on its inner surface, bearing a median-ventral crista (Fig. 3C); crista narrow, turbinate-shaped, with apex protruded to dorsal margin of valve, and with little spines on surface (Fig. 3B), with wider prolongation on the proximal ventral surface connecting with saccus (Fig. 3A). Saccus short, with anterior process curved upwards and thinner apex. Tegumen long and wide, in dorsal view the basal portion of the uncus is wider, narrowing towards the tip, ending in a narrow apical process. Gnathos present, well developed, short tongue-shaped slightly up curved in lateral view (Fig. 3A), vinculum slim and proximally incurved with dorsal projection. Juxta wide in ventral view with a pointed anterior portion and a widened posterior portion as the shape of two slightly divided lobules (Fig. 3F). Aedeagus straight in lateral view, ~ 1/2 the length of the genital capsule, evenly wide, sclerotized, without cornuti (Fig. 3E).

**Female**: Wingspan 50–52 mm, forewing length 26–27 mm. Very similar to male, but paler, with the most prominent dorsal marks and spots mainly in the forewing apex, where it presents a faint stain between the veins R_1_ to M_1_, absent in males and with a paler background than the rest of the wing (Fig. 1C, D). Abdomen with stink-clubs attached to a lateral fold, dorsally on posterior margin of the eighth sternum, densely covered with elongated, either single or bifid, specialized scales (Fig. 3C).

***Female genitalia***: Eighth segment narrow. Posterior apophysis ~ 1/2 the length of the papilla anales (Fig. 3A). Two signa slightly arched with the proximal tip near the ductus bursae, formed by four or five rows of wide spines (Fig. 3B).

#### Immature stages.

**Figure 5.**
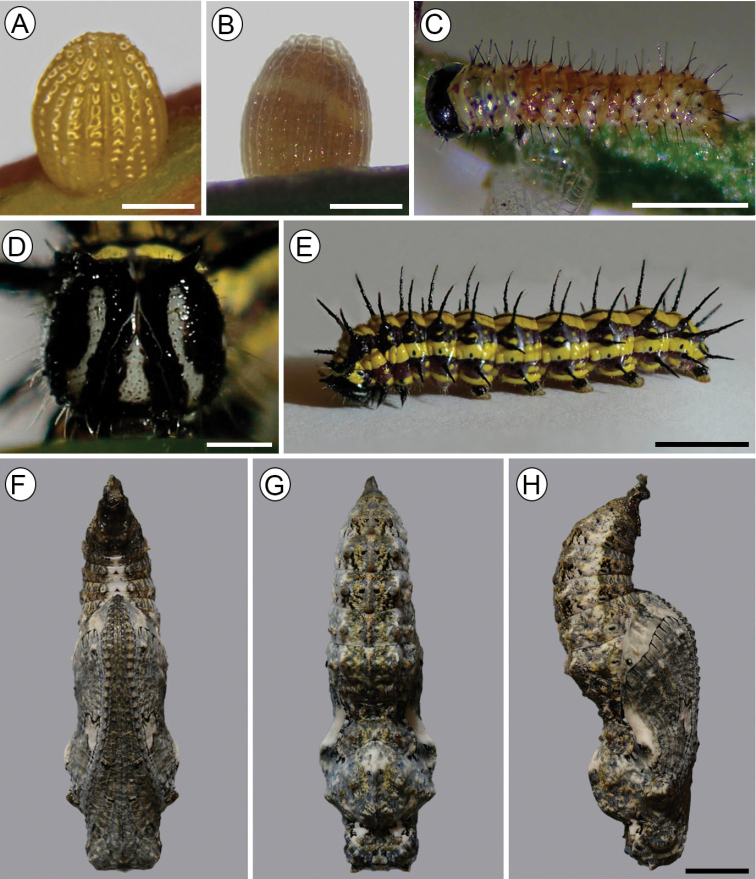
Immature stages of Dione (Agraulis) dodona sp. nov. **A** freshly-laid egg, lateral view **B** egg just prior to hatching, lateral **C** first instar, latero-dorsal **D, E** fifth instar, head in detail (anterior view) and general aspect (lateral), respectively **F, G, H** pupa in ventral, dorsal and lateral views, respectively. Scale bars: 400 µm (**A**); 1 mm (**C**); 5 mm (**E**); 4 mm (**H**).

***Egg*** (Figs 5A, B, 9A, 2S)

Sub spherical, flat base slightly narrowed near apex. Yellow when recently laid (Fig. 5A), reddish brown with a whitish band subsequently, and showing larva by transparency close to hatching (Fig. 5B). Size (mean ± standard error): diameter - 0.92 ± 0.03 mm; height -1.13 ± 0.02 mm (*n* = 10). The chorion is adorned with 19–20 vertical and 16 or 17 horizontal carinae of smooth surfaces, which when intersect delimit cells (Fig. 9A). In the lower part of the egg, the vertical carinae are generally twice as wide as the horizontal ones and some are interrupted at one or two cells before the micropylar region. In the upper third, they have similar widths. Aeropiles scattered in the intersection of carinae, and similar in shape to those of “*Agraulisvanillae*”, as interpreted by da Silva et al. (2006). Micropylar region is surrounded by a rosette-like sculpture of the chorion.

#### First instar.

**Figure 6.**
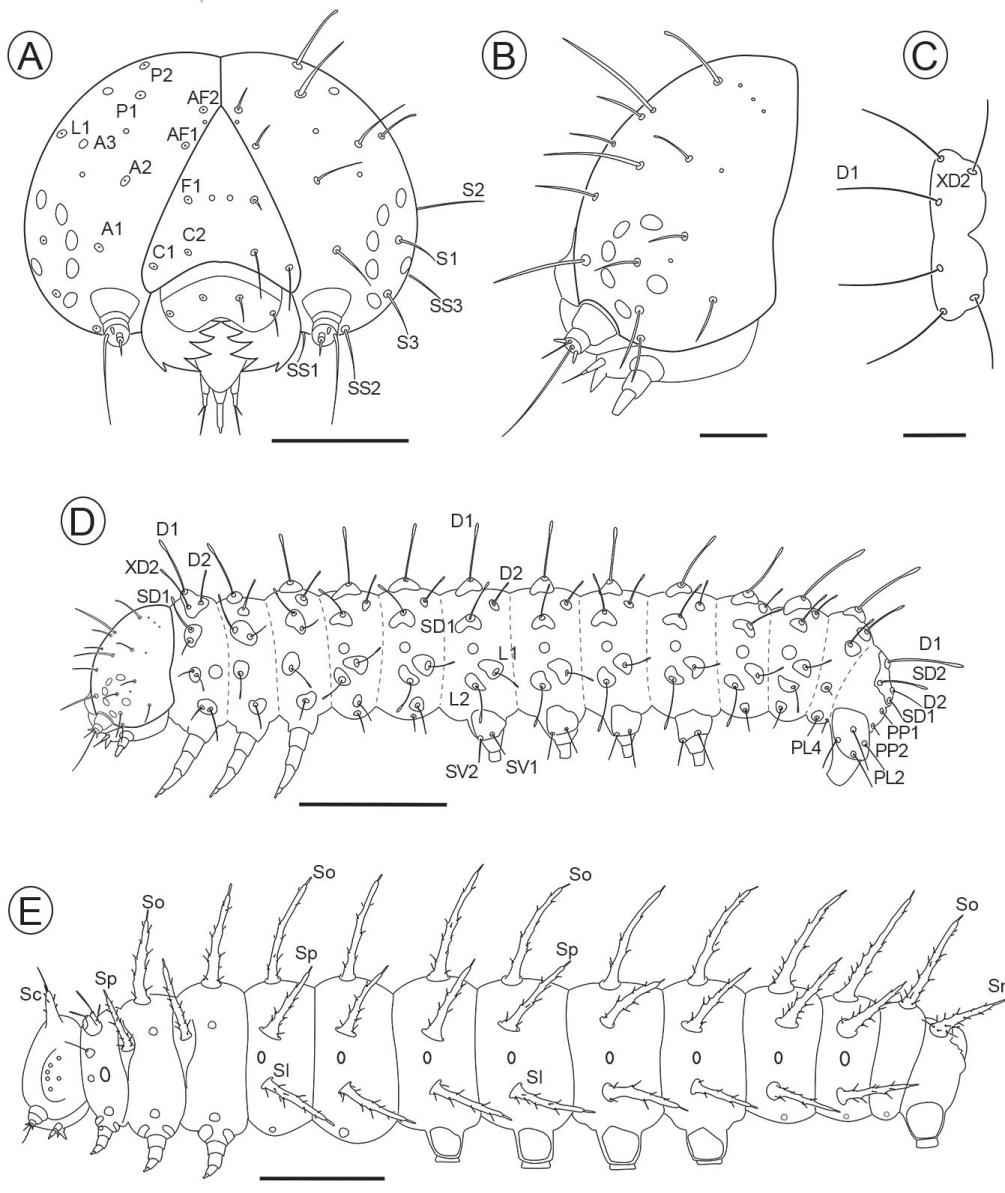
Larval chaetotaxy of Dione (Agraulis) dodona sp. nov. **A, B** head capsule, frontal and lateral view respectively **C** prothoracic dorsal shield, dorsal **D** first instar, lateral **E** fifth instar, lateral. A, anterior seta; AF, adfrontal seta; C, clypeal seta; D, dorsal seta; F, frontal seta; L, lateral seta; P, postero-dorsal seta; S, stemmatal seta; SS, sub-stemmatal seta; PL, seta of proleg cylindrical section of tenth abdominal segment; PP, paraproctal seta; Sc, cephalic scolus; SD, subdorsal seta; Sl, subspiracularscolus; Sn, anal scolus; So, dorsal scolus; Sp, supraspiracularscolus; SV, subventral seta; XD, prothoracic seta. Scale bars: 200 µm (**A**); 100 µm (**B, C**); 500 µm (**D**); 3 mm (**E**).

(Figs 5C, 6A–D, 9B, S2). Length (mean ± standard error; *n* = 6) = 3.56 ± 0.34 mm. Head, prothoracic dorsal shield, anal shield, pinnacles, legs and lateral plates of prolegs blackish; thorax and abdomen mostly creamy white, slightly translucent, reddish brown dorsally on A1–5 (Fig. 5C). Prothoracic dorsal shield trapezoidal with rounded angles, posterior margin with cleft at middle. Chaetotaxy as shown in Fig. 6A–D; SV group unisetose on T2–3; D2 ~ 1/2 the length of D1; and spiracles laterally on prothorax and A1–8, circular, with peritrema elevated (Fig. 9B).

#### Subsequent instars.

**Figure 7.**
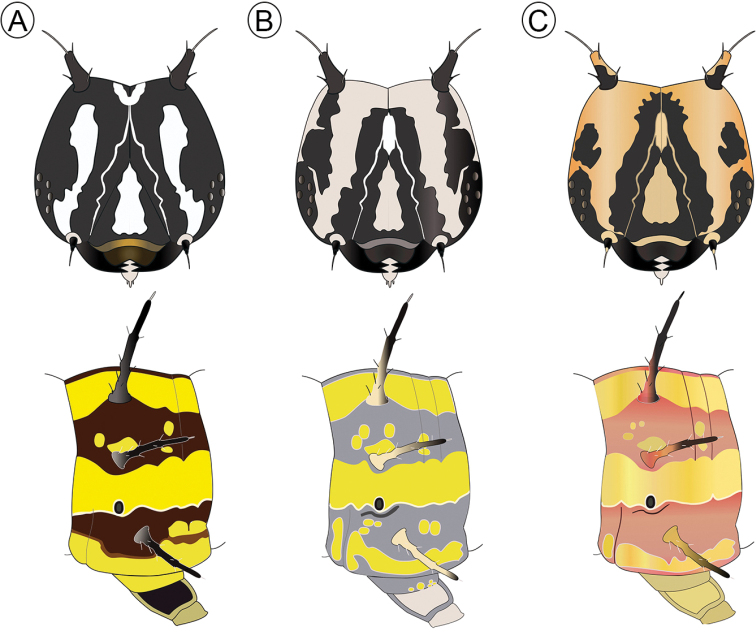
Variation in coloration patterns among fifth-instar of Dione (Agraulis) dodona sp. nov., shown schematically for the head capsule and fourth abdominal segment, respectively, in frontal and lateral views **A** brownish **B** grayish **C** reddish.

From the second instar on, the head is black with thorax and abdomen yellow with two bands running along the subdorsal and subspiracular area. Three chromatic patterns were observed, mainly in the fifth instar, one of these patterns (brownish) is characterized by the head, legs and black scoli; thorax and abdomen yellow with brown band in the subdorsal and subspiracular area, with a thin dorsal brown line, the head has a pattern of white spots located on the frontoclypeous, and with the brown labrum, lateral plates of prolegs black (Fig. 7A); the second colored pattern (greyish) with head and black setae, thorax and abdomen with bands in the subdorsal and subspiracular area gray, the base of prolegs gray too, head with pattern of cream coloration with a larger area than the previous one and the gray labrum, with a triangular spot into the frontoclypeous, lateral plates of prolegs cream (Fig. 7B); the third pattern (reddish) is similar to second pattern but the color of the bands in abdomen, base of scoli and the head is more reddish (Fig. 7C). However, the predominant pattern observed in the field was the brownish one (Fig. 5D, E).

#### Fifth instar.

(Figs 5C, D, 6E, S2) Length (mean ± standard error; *n* = 5) = 25.45 ± 1.78 mm. Head blackish, covered by a large number of hair-like setae and short chalaza-like setae, which vary in length, and bears two short stout scoli dorsally (Fig. 5D). The thorax has the integument covered by conical, striated microtrichia, prominent on coxa of legs and latero-ventral face of prothorax; abdomen with cuticular sculpturing composed of irregular ribs, except the last segment ventrally on posterior face of anal proleg with conspicuous microtrichia; prothoracic dorsal shield bears a number of short chalaza-like setae and two pairs of stout spine-like setae on dorsal conical projections; elliptical spiracles with pronounced peritrema laterally on prothorax and abdominal segments A1–8, those of prothorax and A8 slightly larger than the remaining ones; prolegs with lateral plates covered by several hair-like and chalaza-like setae, crochets in uniserial and multiordinal arrangement. Thirty pairs of thoracic and abdominal scoli, which are elongated conical, integumentary outgrowths, provided with some short chalaza-like setae on the surface, one of which, typically the longest one, is placed at the apex; 11 dorsal pairs (T2–3 and A1–9); ten supra-spiracular pairs (T2–3 and A1–8), with those of the meso- and metathorax anteriorly displaced; eight lateral pairs (A1–8), and one anal pair (A10). Twelve pairs of thoracic and abdominal verrucae; three pairs on prothorax, one between dorsal shield and spiracle, which is provided with a spine-like seta, another greatly reduced pair anterior to spiracle, and another pair between spiracle and coxa; two pairs on meso and metathorax, one posterior to supraspiracular scolus and another dorsal to coxa; one pair on A1–2 and A7–8, which is ventral to lateral scolus; and one pair on A9, on the latero-ventral face of the segment.

#### Instar identification.

The successive instars can be accurately distinguished by the width of the head capsule, because they do not overlap (Table 2). The corresponding exponential growth equation was adjusted for the five instars: y = 0.328eˆ0.403x; *n* = 62; r^2^ = 0.980; p < 0.05. Thus, the growth pattern of the head capsule follows the Brooks-Dyar´s rule (Daly 1985). The mean growth ratio among instars was 1.495, similar to ratios previously reported for other Neotropical heliconians (Antunes et al. 2002; Kaminski et al. 2002, 2008; Tavares et al. 2002; Paim et al. 2004; da Silva et al. 2006, 2008; Barão and Moreira 2010; Vargas et al. 2014; Barão et al. 2015).

**Table 2.** Mean and standard error (SE), interval of variation (IV), and growth rates (GR) of head capsule width in larval instars of Dione (Agraulis) dodona**sp. nov**. reared on *Malesherbiatenuifolia*D. Don.

Instar	N	Head capsule width (mm)		
		Mean ± SE	IV	GR
I	19	0.50 ± 0.01	0.46–0.56	-
II	12	0.72 ± 0.01	0.58–0.83	1.44
III	8	1.07 ± 0.03	0.95–1.17	1.48
IV	8	1.69 ± 0.04	1.56–1.87	1.52
V	5	2.50 ± 0.05	2.44–2.70	1.48
				

**Figure 8.**
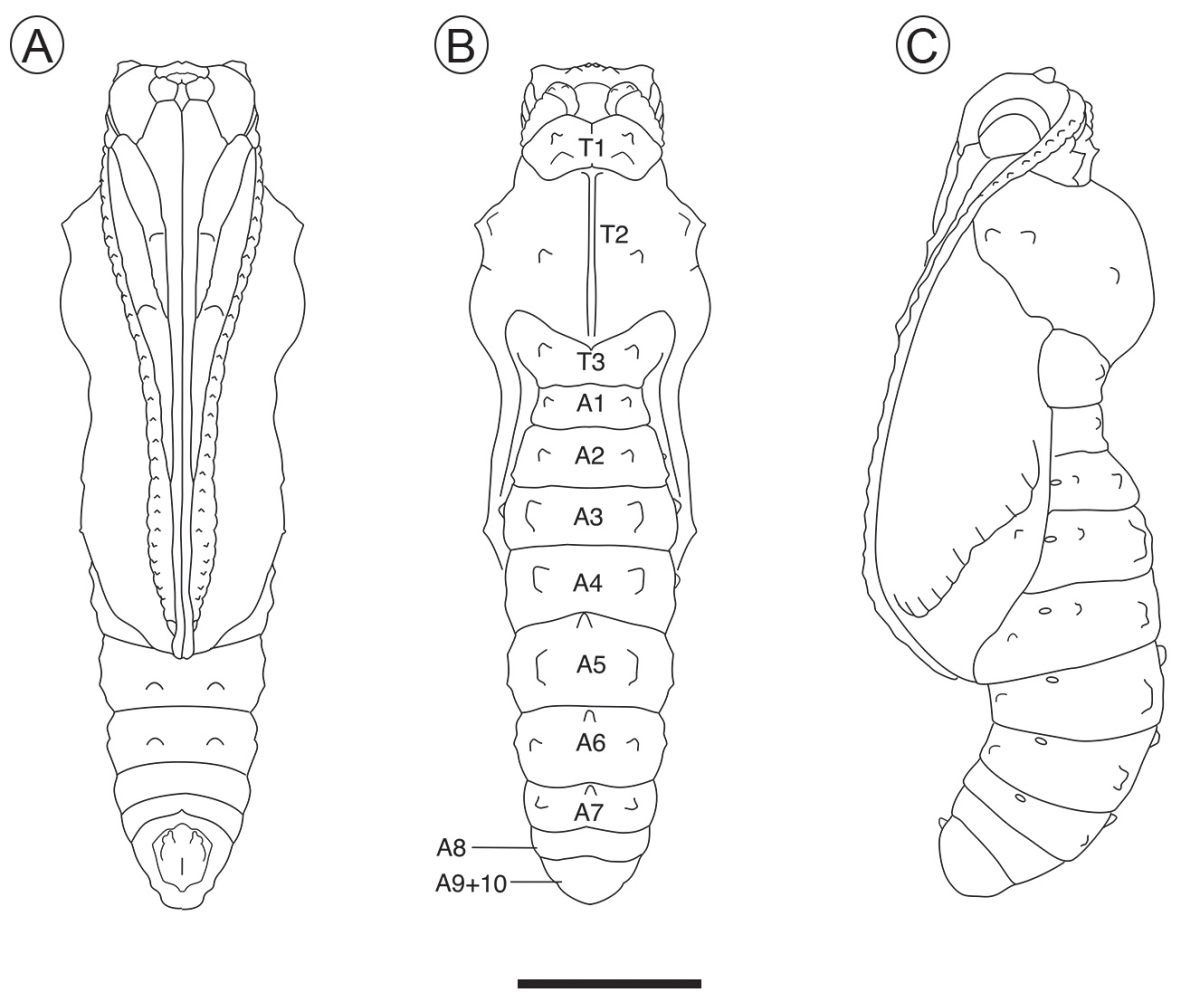
Pupa of Dione (Agraulis) dodona sp. nov. under **A** ventral **B** dorsal, and **C** lateral view, respectively. A, abdominal segment; T, thoracic segment. Scale bar: 4 mm.

**Figure 9.**
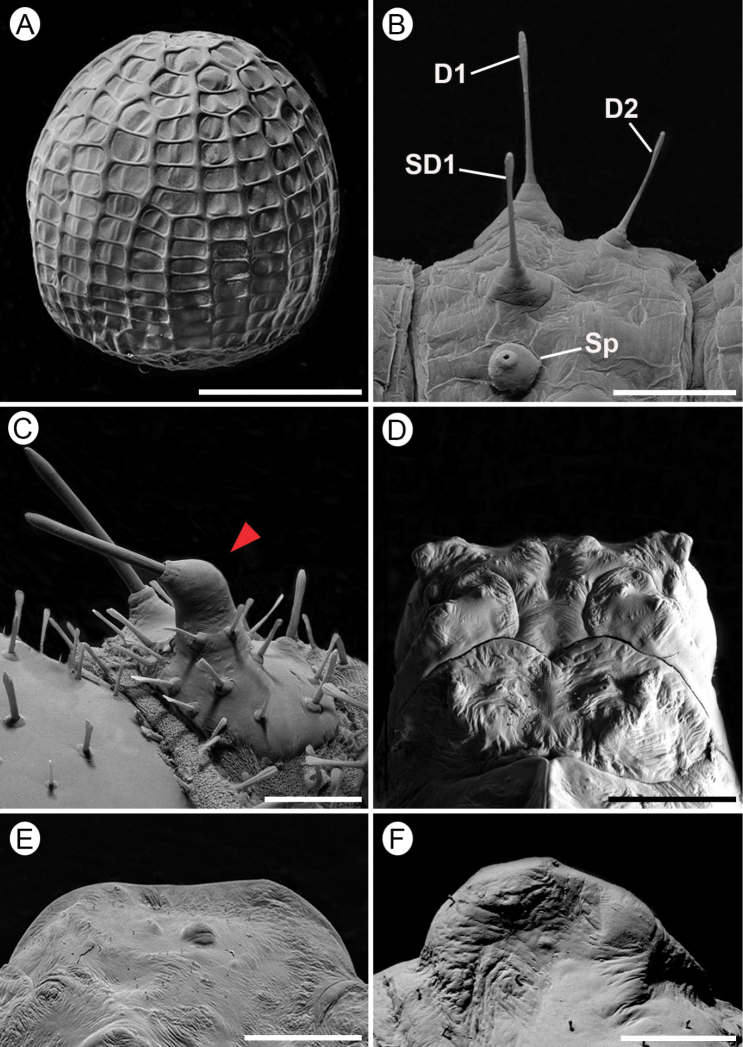
Immature stages of Dione (Agraulis) dodona sp. nov. under scanning electron microscopy **A** egg, lateral view **B** first instar, upper portion of fifth abdominal segment, lateral **C** fifth instar, prothoracic plate, postero-dorsal (protuberance pointed by close arrow) **D, E, F** pupal head (dorsal view), mesothoracic meso-dorsal crest (lateral), and latero-dorsal tubercle of third abdominal segment (lateral), respectively. Scale bars: 500 µm (**A, F**); 100 µm (**B**); 250 µm (**C**); 1.5 mm (**E, D**).

***Pupa*** (Figs 5F–H, 8, 9D–F) General shape elongated, ground color non-uniform, consisting of a mixture of shades of gray, light brown, and ocher (Fig. 5F–H), with variation between individuals in their intensity. Length (mean ± standard error; *n* = 5) = 19.98 ± 0.28 mm. Head with pair of short, angled cephalic projections (Figs 8, 9D); epicranial suture absent; eyes with sculptured region near antenna, bearing few short hair-like setae, and another smooth region near front; labrum as a slight, short longitudinal stripe between the mandibles; maxilla with well-developed galeae, along midline of ventral surface, anteriorly delimited by labrum and mandibles, slightly surpassing posterior margin of A4; antennae arising laterally on head, projected ventrally to apex of maxilla, with many smooth, round tubercles on surface. Thorax with the three segments exposed. Prothorax as a small hexagonal plate in dorsal view, with anterior and posterior margins broadly excavated, with two pair of lateral tubercles. Mesothorax broadly expanded laterally along anterior half with a meso-dorsal crest that is well developed, broadly rounded, bearing a pair of round lateral tubercles; two pairs of marginal and submarginal tubercles close to base of wings; one pair of submarginal tubercles each near apex of wing; mesothoracic spiracle opening laterally at anterior margin of segment. Metathorax as a narrow plate with anterior margin broadly excavated, with pair of lateral tubercles, hindwings as straight stripes between forewings and abdominal segments. Abdomen with segments A1–A4 partially hidden by wings; with pair of lateral tubercles on A1–A7 which are little developed on A1–A2, most developed on A3, and decreasing in size posteriorly; one meso-dorsal tubercle on A5–A7; one supraspiracular tubercle on A2–A4; one pair of subspiracular tubercle on A4, pair of ventral tubercles on A5–A6; spiracles of A1 and A2 hidden and partially hidden, respectively, by forewings, and spiracles of A3–A7 elliptical; anal segment with two prominent tubercles, ventrally. Cremaster quadrate, with truncate apex, and a large number of short, curved hooks.

#### Etymology.

The specific epithet is based on the locality of Dodona (Greece); it was a city-sanctuary in ancient Greece, where there was an oracle in which Dione was venerated as the (temporary) wife of Zeus, until she was replaced by Hera. Thus, the new species is named “dodona” to continue the classical Greek tradition.

#### Distribution.

Adults of Dione (Agraulis) dodona sp. nov. are known from distinct populations, located in central and southern Peru, and northern Chile, on the western slopes of the Andes. In Peru, it has been found in the Departments of Lima (1,400–2,400 m elevation), Arequipa (between 800 and 2,600 m elevation), Moquegua (1,800–2,100 m elevation), and Tacna (1,800 m elevation). In Chile, two specimens were collected in 1951 and 1968 from two localities in the Tarapacá Region between 2,300 and 3,000 m elevation, and recently other two specimens were collected from Arica Region (1,580 m elevation).

#### Host plant.

**Figure 10.**
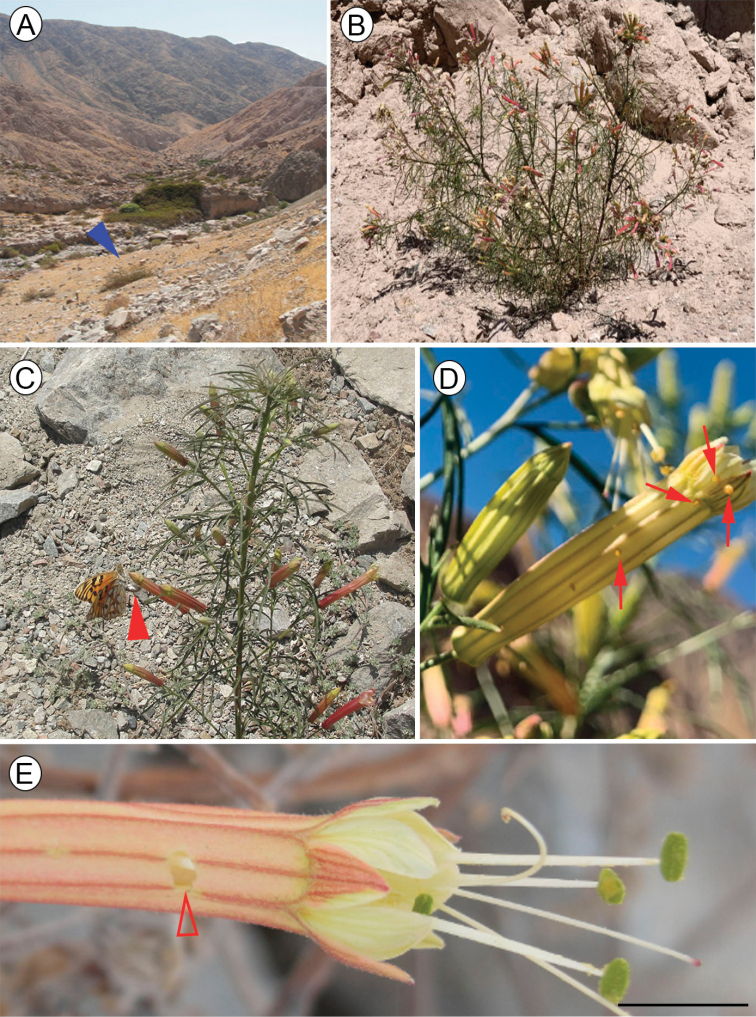
Life history of Dione (Agraulis) dodona sp. nov. **A** general view of type locality, Pacaychacra valley, Arequipa, Peru (blue close arrow points to larval hostplant) **B** host plant, *Malesherbiatenuifolia* Don **C** female laying eggs (close arrow point) on flowers of host plant **D** eggs (pointed by setae) on flower **E** detail of flower showing damage (entrance) by first instar larva (open arrow). Scale bar: 5 mm.

*Malesherbiatenuifolia*D. Don (Passifloraceae) is the only host plant known for the immature stages of Dione (Agraulis) dodona sp. nov. This species was described originally from northern Chile. It is distributed between 19–21 °S in Chile (Bull-Hereñu 2020), and was reported recently from southern Peru (Weigend et al. 2015; Beltrán et al. 2018), restricted to the western slopes of the Andes above 1,500 m. *M.tenuifolia* is a shrub with reddish or yellowish tubular flowers that grows to ca. 1 m in height (Fig. 10), associated with the dry beds and immediate surroundings of seasonal rivers in Chile (Bull-Hereñu 2020), and steep scree slopes along the road cuts in the type locality of Dione (Agraulis) dodona sp. nov. (Fig. 10A).

#### Life history.

Adults of Dione (Agraulis) dodona sp. nov. were only observed on sunny days in the type locality, beginning to fly around 08:00 a.m., quickly disappearing when weather conditions became cloudy. They usually fly close to the ground, up to 1–2 m high. Females were seen alighting on the host plant. Territorial behavior, courtship and mating behavior were not observed. Adults were seen feeding on flowers of other plants growing around *Malesherbia* hosts. The species is multivoltine in the population of the type locality, flying all year round. From three to seven individuals were usually observed in a typical sunny day (4–5 h of observation), most of which were males. This species is sympatric with Dione (Agraulis) forbesi, but they do not fly in the same habitat; *forbesi* was observed in areas with higher humidity and vegetation, compared to *dodona*, which was found only in xerophytic areas associated with the host plant (Fig. 10A, B). Females were observed laying eggs particularly on flowers (Fig. 10D). Oviposition occurred with the female sitting on top of the flower and curling the abdomen around the flower edge so that eggs were deposited underneath (Fig. 10C). During visits made at different months of the year in the type locality, freshly laid eggs were always collected, both on flowers and leaves. Between one and eight eggs were found in the same plant, in different flowers or leaves, but sometimes several eggs were obtained from the same flower (Fig. 10D). The eggs are laid isolated from each other. Newly hatched larvae first consumed the chorion, and afterwards began to feed inside of flower, leaving a hole through which they enter the flower (Fig. 10E). Subsequent instars feed externally on leaves. Larvae were consistently solitary in all instars, regarding all activities, such as feeding or resting. Pupae were found predominantly off the host plant, clinging to rocks near the host plant, and sometimes on branches of the host plant close to the ground.

#### Molecular data.

**Figure 11.**
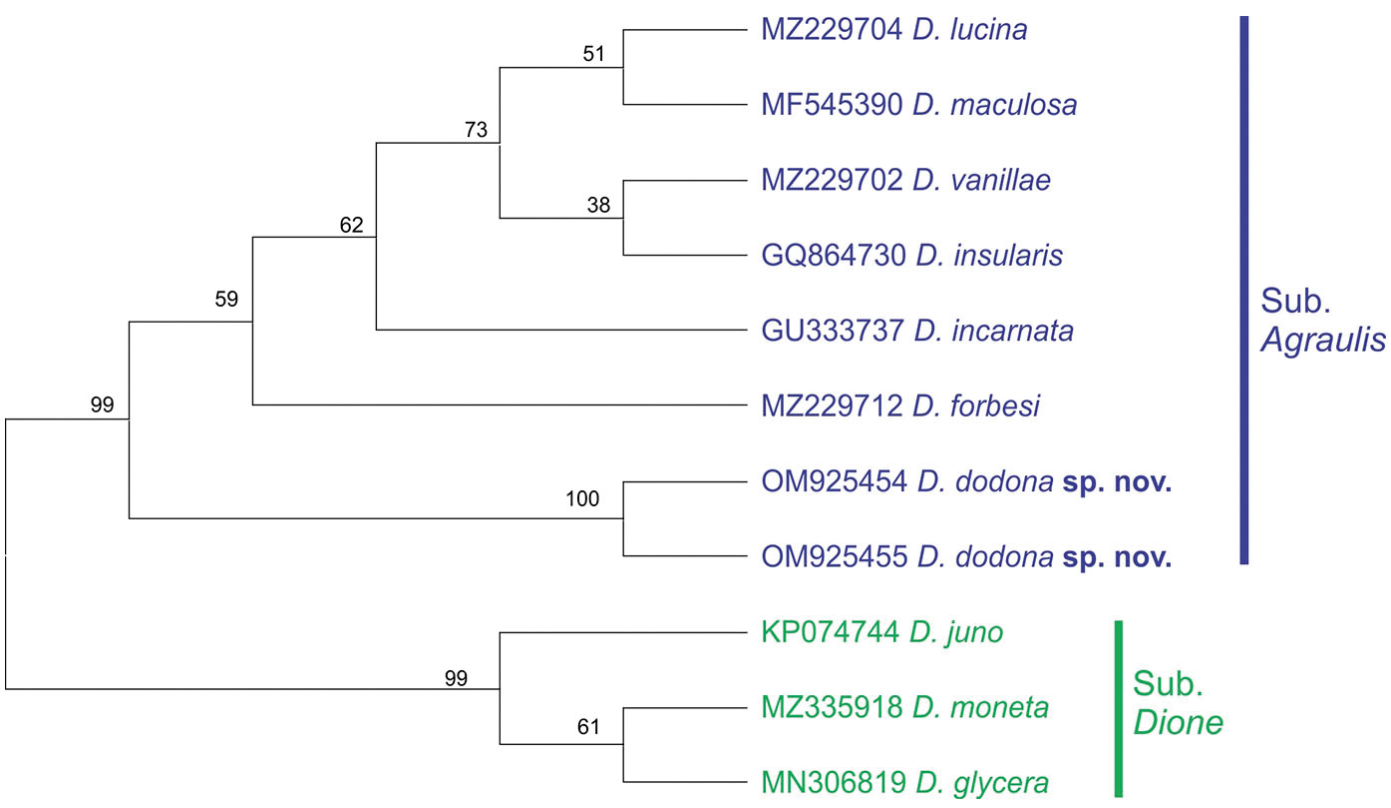
Phylogenetic status of Dione (Agraulis) dodona sp. nov. based on 650 bp-sequences of the Cytochrome oxidase subunit I gene. The consensus tree was inferred with the Maximum Likelihood method and General Time Reversible model, with 500 bootstrap replicates. The branch support (bootstrap) is shown next to the nodes.

Dione (Agraulis) dodona sp. nov. was recovered as an independent lineage within the *Agraulis* clade of the COI-tree (Fig. 11), diverging in ca. 5% to the group formed by its other species, and 9.5% to the *Dione* clade (Table 3).

**Table 3.** Genetic distance (%) between Dione (Agraulis) dodona sp. nov. and its congeners. Analysis used 650 base pairs sequences of the Cytochrome oxidase subunit I gene under the Kimura 2-parameter model. Specimens included in the analysis are presented in Table 1.

Taxa	1.	2.	3.	4.	5.	6.	7.	8.
1. Dione (Agraulis) dodona sp. nov.	-							
2. Dione (Agraulis) forbesi	5.8	-						
3. Dione (Agraulis) lucina	4.6	3.9	-					
4. Dione (Agraulis) vanillae	5.3	4.4	2.2	-				
5. Dione (Agraulis) incarnata	5.1	4.6	3.4	3.4	-			
6. Dione (Agraulis) insularis	4.6	3.9	1.9	1.9	3.2	-		
7. Dione (Agraulis) maculosa	4.4	3.4	1.1	1.7	2.7	1.1	-	
8. Dione (Dione) spp.	9.5	8.9	8.6	8.6	9.3	9.4	8.8	-

## ﻿Discussion

The identification key to adults of *Agraulis* provided by Núñez et al. (2022) based on external morphology, indicated only one character of the wing pattern color to separate Dione (Agraulis) dodona sp. nov. (listed as *Agraulis* sp.), an extra black spot on the upper hindwing cell Cu_1_-Cu_2_ (Fig. 1E). As already mentioned, we propose in addition the divided silver discal spot on the hindwing underside (Fig. 1F); this last trait is not found in other species of the *Agraulis* clade, but is shared with other species of *Dione*. Núñez et al. (2022) found that Dione (Agraulis) galapagensis W. Holland, 1890 and Dione (Agraulis) dodona sp. nov. have similar size (forewing length 27 mm or less) but are smaller in relation to other species of the *Agraulis* clade. These findings are supported by the specimens studied by us, where the longest wings of Dione (Agraulis) dodona sp. nov. were from a male and a female, reaching up to 28 and 27 mm, respectively; however, this should not be a morphological attribute to be used to separate species. Regarding the male and female genitalia, Dione (Agraulis) dodona sp. nov. are very distinct from those of other members of the *Agraulis* clade, the most notable differences being in the valval termen without denticles, the narrow crista projection and the shape of yuxta in males (see Suppl. material 1), as well as the shape of the signum in females. According to Emsley (1963), these characters have an important value to differentiate among species within Heliconiinae. Thus, morphological characters of adults described for Dione (Agraulis) dodona sp. nov. allow its clear differentiation from other species. Similarly, phylogenetic relationships inferred from DNA sequence data support Dione (Agraulis) dodona sp. nov. as a distinct taxon, placed as external to the group formed by its congeners, considered the earliest diverging (ca. 8 Mya) lineage within the *Agraulis* clade (Núñez et al. 2022). Although sharing morphological traits with the *Dione* clade, the genetic distance of nearly 10% is quite high, which together with previous inferences using several molecular markers (Kozak et al. 2015; Massardo et al. 2015), supports the recognition of two evolutionary lineages that shared a common ancestor around 14 million years ago (Núñez et al. 2022). Following Zhang et al. (2019), we consider that the phylogenetically most informative hypothesis is to treat *Dione* and *Agraulis* as equivalent subgenera. In summary, as previously accepted by Núñez et al. (2022), Dione (Agraulis) dodona sp. nov. is evidenced as a valid species based on morphological and molecular evidence.

Thus, results presented herein show clearly that Dione (Agraulis) dodona sp. nov. is distinct from its congeneric species at all development levels. On the other hand, particularly in the larval stage, our study also showed that it shares some characters with species of the *Dione* clade; in the first instar, the D2 setae are well developed accordingly in the latter; in subsequent instars, the cephalic scoli are reduced similar with *Dionemoneta*, and the prothoracic plate with enlarged conical protuberances bears setae. In fact, such traits had been used up to now to separate genera of heliconiines at the larval stage (Fleming 1960; Tavares et al. 2002; Kaminski et al. 2008). Also, Dione (Agraulis) dodona sp. nov. exhibits some unique characters in the fifth instar that were not observed in other species in the genus *Dione* s. l. Among them, it is worth highlighting the spiracles with pronounced peritrema, the predominance of short chalaza-like setae covering abdominal segments including scoli, and the linear distance between stemmata I and II larger than distance between stemmata II and III in head (see Suppl. material 2). Since the phylogenetic relationships between the *Agraulis* and *Dione* clades and their taxonomic consequences have been controversial, we suggest to explore the evolutionary history of these genera taking into account morphological characters herein found for Dione (Agraulis) dodona sp. nov. within a cladistic approach.

Females of Dione (Agraulis) dodona sp. nov. lay eggs predominantly on flowers, where first instar feed on the internal parts. There is no documentation of another species of Heliconiinae that oviposits preferentially on flowers or larvae feeding on them (de Castro et al. 2018). The host plant of Dione (Agraulis) dodona sp. nov., *Malesherbiatenuifolia*, represents the first record of the genus as a host plant in Heliconiinae (Beccaloni et al. 2008). This genus represents one of the oldest lineages of the Passifloraceae s.l. (Tokuoka 2012), considered until a few years ago as a separate family (Malesherbiaceae) (Gengler-Nowak 2003), but recent phylogenetic studies proposed to include it in Passilforaceae s. l. (Soltis et al. 2007; Bremer et al. 2009). *Malesherbia* are a little-known group of xerophytic plants endemic to a variety of arid habitats in the Pacific coastal desert and adjacent Andes of Peru, Chile, and neighboring Argentina (Gengler-Nowak 2002; Bull-Hereñu 2020). The subgeneric classification of *Malesherbia* comprises five sections (Gengler-Nowak 2003), *M.tenuifolia* belongs to the *Malesherbia* section Gengler-Nowak (2003: 343) along with other species that are distributed at the northern limit of the genus, from central Peru to northern Chile (Gengler-Nowak 2003; Beltrán et al. 2018; Bull-Hereñu 2020). Coincidentally, the known populations of Dione (Agraulis) dodona sp. nov. follow the geographical distribution of this section of *Malesherbia*, and this could imply that Dione (Agraulis) dodona sp. nov. uses other *Malesherbia* species as hostplants, considering that in the Department of Lima (central Peru) where Dione (Agraulis) dodona sp. nov. was collected, four *Malesherbia* species have been reported, but not *M.tenuifolia* (Beltrán et al. 2018).

Finally, it is important to mention that, historically, Dione (Agraulis) dodona sp. nov. was erroneously cited for decades as “*Agraulisvanillae*” in Chile, based on two specimens collected between 1950 and 1970 in the Tarapacá region, northern Chile. Peña (1951) reported it for the first time in Chile based on a male specimen collected in the locality of Parca, and afterwards Pérez-D’Angello (1970) reported a second specimen from Guatacondo, in the collection of Pedro Millas. Subsequently, all Chilean butterfly checklists published cited “*A.vanillae*” as a species distributed in northern Chile (Ureta 1963; Etcheverry 1970; Herrera 1972; Peña and Ugarte 2006; Benyamini et al. 2014). By examining both specimens that are currently at MUSM (the second one on loan from MHNS), we confirm their identity as Dione (Agraulis) dodona sp. nov. Probably, the host plant used by this population from northern Chile is *M.tenuifolia*, reported in these localities of Tarapacá region (Bull-Hereñu 2020).

## Supplementary Material

XML Treatment for Dione (Agraulis) dodona

## Supplementary materials

Supplementary material 1 Figure S1 Data type Image. Explanation note Male genitalia of Dione (Agraulis) species congeners to Dione (Agraulis) dodona sp. nov. (A) *D.* (*A*) *galapagensis*; (B) D. (A.) lucina; (C) D. (A.) maculosa; (D) D. (A.) vanillae; (E) D. (A.) forbesi; (F) D. (A.) insularis; (G) D. (A.) incarnata incarnata; (H) D. (A.) incarnata nigrior. From right to left column: General, mesal view, aedeagus removed; termen of valve, mesal; juxta, ventral; aedeagus, lateral. Scale bars: 1mm, 0.25mm, 0.25mm, 0.5mm, respectively. https://binary.pensoft.net/file/716559This dataset is made available under the Open Database License (http://opendatacommons.org/licenses/odbl/1.0/). The Open Database License (ODbL) is a license agreement intended to allow users to freely share, modify, and use this Dataset while maintaining this same freedom for others, provided that the original source and author(s) are credited.Jackie Farfán

Supplementary material 2 Figure S2 Data type Image. Explanation note Scanning electron micrographs of Dione (Agraulis) dodona sp. nov. immature stages: (A-C) Egg; (D-J) First instar; (K-P) Fifth instar. (A) micropylar region; (B) aeropyle; (C) upper cells; (D) stemmatal region, lateral; (E) antenna, antero-lateral; (F) spinneret, lateral; (G) metathoracic leg; (H) apex of setae of abdominal segment, lateral; (I) proleg with exposed crochets; (J) last abdominal segment, posterior; (K) stemmatal region, dorso-frontal; (L) short chalaza-like setae; (M) proleg of six abdominal segments; (N) seventh abdominal segment, latero-dorsal; (O) spiracle; (P) corresponding spiracle in detail. Mp, micropyles; Ac, aeropyle; Uc, upper cell. Scale bars: 50 µm (A, E, F, I, L, Q); 10 µm (B); 150 µm (C, P); 100 µm (D, G, J); 25 µm (H, M); 250 µm (K); 0,5 mm (N); 1mm (O). https://binary.pensoft.net/file/716560This dataset is made available under the Open Database License (http://opendatacommons.org/licenses/odbl/1.0/). The Open Database License (ODbL) is a license agreement intended to allow users to freely share, modify, and use this Dataset while maintaining this same freedom for others, provided that the original source and author(s) are credited.Jackie Farfán
