# Supplementary figures and images for: ﻿A peculiar new species of Dione (Agraulis) Boisduval & Le Conte (Lepidoptera, Nymphalidae, Heliconiinae) associated with Malesherbia Ruiz & Pavón (Passifloraceae) in xeric western slopes of the Andes
Source: Zookeys. 2022 Jul 18;1113:199–226. doi: 10.3897/zookeys.1113.85769 (PMC9848672; doi:10.3897/zookeys.1113.85769)

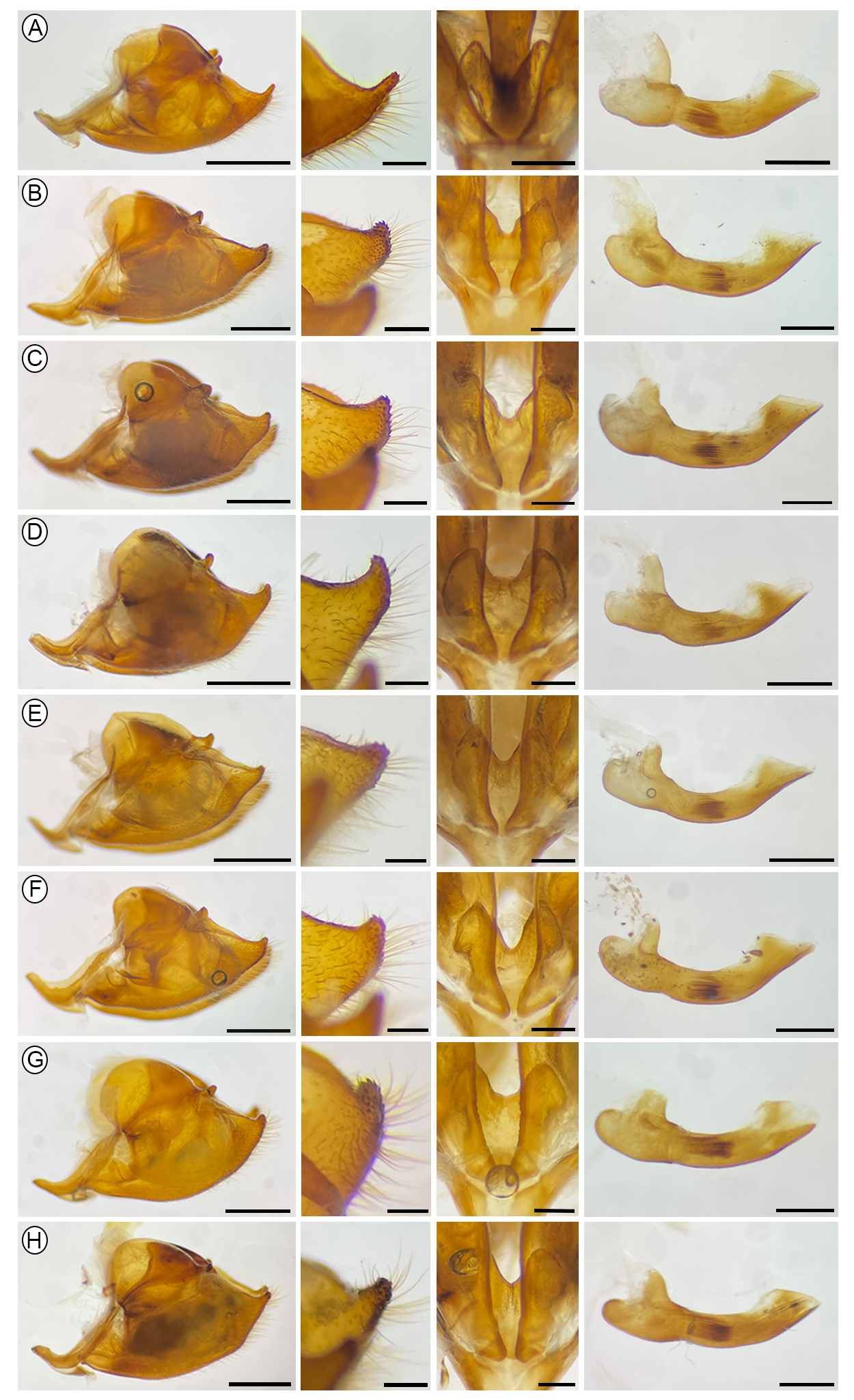

Supplement: Supplementary material 1 — Figure S1 [file zookeys-1113-199_article-85769__-s001.jpg]

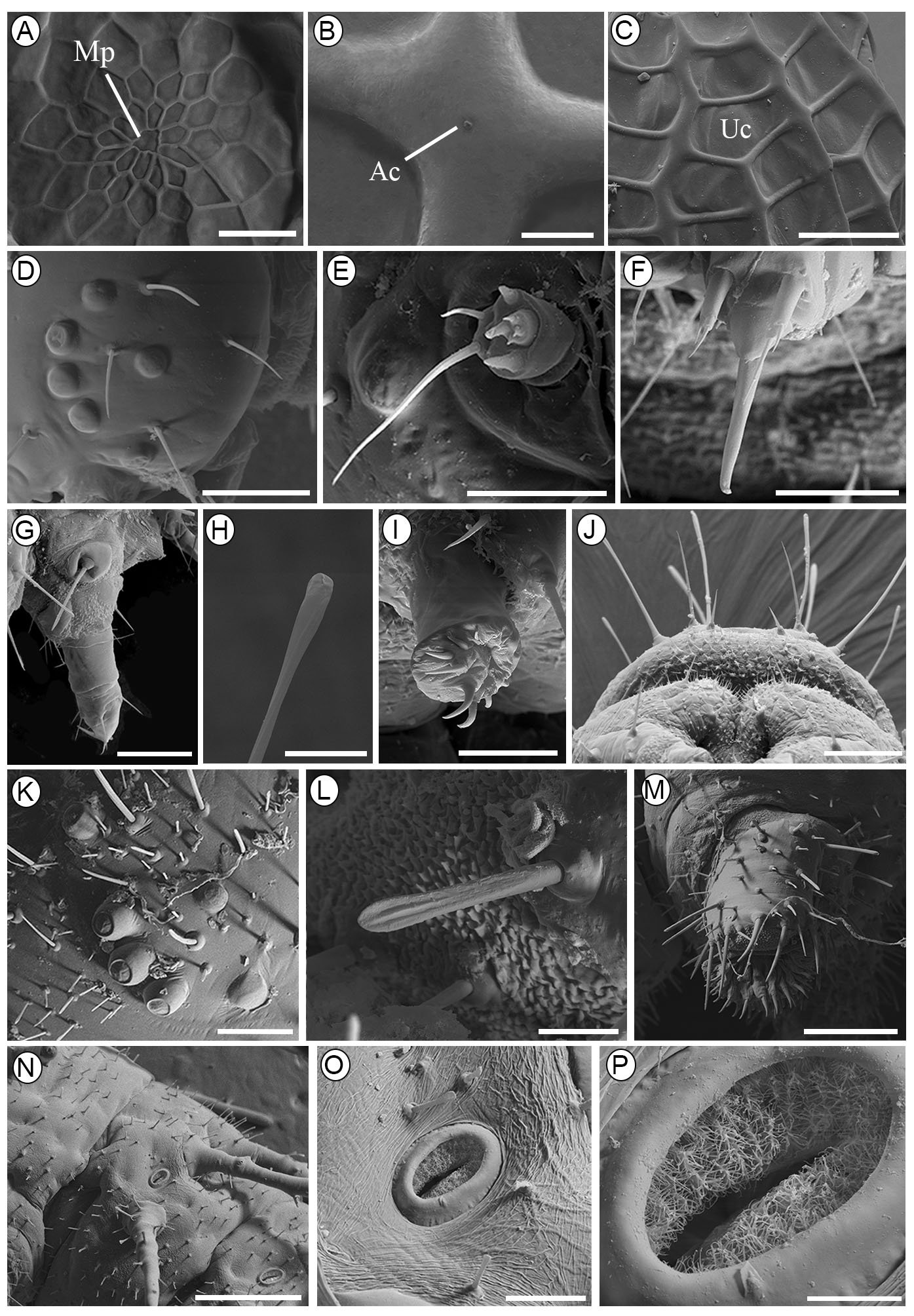

Supplement: Supplementary material 2 — Figure S2 [file zookeys-1113-199_article-85769__-s002.jpg]
